# *Entada africana* fraction CH_2_Cl_2_/MEOH 5% inhibits inducible nitric oxide synthase and pro-inflammatory cytokines gene expression induced by lipopolysaccharide in microglia

**DOI:** 10.1186/1472-6882-13-254

**Published:** 2013-10-03

**Authors:** Brice Ayissi Owona, Nico Frederic Njayou, Stefan A Laufer, Hermann J Schluesener, Paul Fewou Moundipa

**Affiliations:** 1 Division of Immunopathology of the Nervous System, Department of Neuropathology, Institute of pathology, Calwerstraße 3, Eberhard-Karls-Universität, Tuebingen, Germany; 2 Laboratory of Pharmacology and Toxicology, Department of Biochemistry, University of Yaoundé 1, Yaoundé 1, Cameroon; 3 Medical Chemistry Department, Pharmacy & Biochemistry, Eberhard-Karls-Universität Tuebingen, Auf der Morgenstelle 8, Tuebingen, 72076, Germany

**Keywords:** *Entada africana*, Baicalin, Microglia, Cytokines, p38 MAPK Kinase

## Abstract

**Background:**

Inflammatory response in the CNS mediated by microglia cells play an important role in host defense and is implicated in the pathology of neurodegenerative diseases. We investigated the capacity of *Entada africana* to protect microglia from inflammatory insults by exploring the effect of the CH_2_Cl_2_/MEOH 5% fraction (Ea5) on pro-inflammatory cytokines mRNA expression. Finally, we studied the effect of Ea5 on the inhibition of p38 MAPK Kinase. The results were compared to those obtained with Baicalin, a well reported anti-inflammatory flavonoid.

**Methods:**

Barks from *E. africana* were harvested in 2010, in the west region of Cameroon. A crude extract was prepared using CH_2_Cl_2_/MEOH 1:1 V/V. The crude extract obtained was further fractionated by flash chromatography. A mouse microglia cell line (N9) was stimulated by LPS with or without different concentrations of Baicalin and Ea5. The release of NO was evaluated using the Griess method. The expression of pro-inflammatory cytokines mRNA (TNFα, IL-1β, IL-6) and iNOS/NO were measured by RT- PCR. The inhibition of p38 MAPK Kinase was assessed using ELISA.

**Results:**

We found that Ea5*, as* well as Baicalin inhibited LPS-induced NO production in a dose dependent manner. Ea5 was most active in term of NO inhibition (87.07%), in comparison to Baicalin (70.85%). The expression of TNFα, IL-1β, IL-6 and iNOS was strongly suppressed by Ea5 in microglia. Ea5 also inhibited the activity of p38MAPK Kinase, up to 30% for the concentrations tested, whereas a prominent inhibition was obtained with Baicalin.

**Conclusion:**

These results suggest that *E. africana* may contain promising compounds useful for the treatment of diseases cause by over-activation of microglia such as Alzheimer disease and other neurological diseases.

## Background

Inflammation has recently been implicated as a critical mechanism responsible for the progression of neurodegenerative disorders [1]. Microglia, the resident macrophage-like cells in the brain are reported to produce a barrage of elements (IL-1, TNFα, NO, PGE_2_, superoxide) that are toxic to neurons [2]. Microglia have a critical role in host defense against invading microorganisms and their actions appear to influence neuronal proliferation, as well as contribute to the removal of dying neurons or cellular debris [3]. The effects of microglia on the central nervous system can be ascribed to the numerous substances that these cells can synthesize and release in response to a variety of stimuli (cytokines, pro-inflammatory substances, toxins, etc.) [4]. Moreover, microglia activation is associated with the pathogenesis and progression of diseases such as Alzheimer’s disease (AD), Parkinson’s’ disease, multiple sclerosis, and traumatic brain injury [5].

Mitogen-activated protein kinases (MAPKinase) are intracellular signaling kinases activated by phosphorylation in response to a variety of extracellular stimuli [6]. Inhibitors of p38 MAP kinase are considered as suitable targets in the treatment of inflammatory diseases [7]. As a result of these observations, many inhibitors of p38 MAPKinase are more and more investigated for their anti-inflammatory effects [8,9].

Therapeutic approaches focused on inhibition of the microglia-mediated local inflammatory response in the brain may offer new opportunities to treat neurological diseases. Amongst them, Baicalin (BA) is a flavonoid compound purified from the Chinese medicinal plant *Scutellaria baicalensis Georgi*. Baicalin regulated Toll-like receptor 2/4 after ischemic neuronal injury and the inflammatory reaction in neuron damage [10]. Baicalin improved survival in a murine model of polymicrobial sepsis via suppressing inflammatory response and lymphocyte apoptosis [11]. Baicalin attenuated inflammation by inhibiting NF-kappaB activation in cigarette smoke induced inflammatory models [12]. Many *in vitro* studies showed that Baicalin suppressed the increased generation of nitric oxide (NO) and expression of inducible nitric oxide synthase (iNOS) induced by LPS [13].

*E. africana* (Guill. & Perr, Family: Fabaceae) is used in African traditional medicine for the treatment of many diseases. The plant is used in Mali for the treatment of Malaria [14]. Anti-inflammatory, hepatoprotective and wound healing effects have also been demonstrated [15]. In Burkina Faso, the plant is used for the treatment of diabetes, hypertension and diarrhea. In Cameroon, the plant is used for the treatment of wound dressing, fever, liver related diseases, wound healing, rheumatism, cataract, fevers and dysentery. Studies on *E. africana* concern antimicrobial, antiplasmodial and antioxidant activities [16], fungistatic, fungicidal [17] and anti-ulcerogenic activities [18].

In a recent article published by our research group, we showed that *E. africana* fraction CH_2_Cl_2/_MeOH 1.1 V/V 5% (Ea5) suppresses lipopolysaccharide-induced inflammation in Raw 264.7 macrophages [19]. Here we investigated the effect of Ea5 on NO production and the expression of pro-inflammatory cytokines mRNA by microglia in response to LPS. We also examined the effect of Ea5 and Baicalin on iNOS mRNA expression and on p38 MAP Kinase inhibition.

## Methods

### Plant materials and solvent extraction

*E. africana* was harvested in 2010, in the West region of Cameroon. The Voucher specimen was identified by Dr Njayou Frederic Nico of the University of Yaoundé I, Cameroon, and deposited at the National Herbarium, Yaoundé, Cameroon (ID: 244366, Voucher number: 52661 YA). Dried *E. africana* barks were air dried, cut into small pieces and ground. One Kg of the powder was immersed and extracted in methylene chloride/methanol 1/1 v/v at room temperature for 7 days. After the mixture was filtered, the filtered cakes were extracted and filtered three more times to increase the extraction yield. The procedure was repeated until the solvent present a clear color. The filtrate was concentrated under reduced pressure, and the crude extract obtained was freeze-dried, and stored at 4°C until used. The crude extract was subjected to flash chromatography to obtain the Ea5 fraction (CH_2_Cl_2_/MeOH 5%).

### Chemicals

Fetal bovine serum (FBS), antibiotics (streptomycin/penicillin), and RPMI medium were purchased from Gibco (Grand Island, NY, USA). Escherichia coli-LPS and 3-(4, 5- dimethylthiazol-2-yl) -2, 5- diphenyltetrazolium bromide (MTT) were purchased from Sigma-Aldrich (St. Louis, MO, USA). Baicalin (99%) was purchased from Carbosynth Ltd. (Compton, Berkshire, UK). Mouse cytokines primers (iNOS, TNFα, IL-6, and IL-1β) were supplied by Santa Cruz Biotechnology (Santa Cruz, CA, USA).

### In vitro cell culture

The microglia cell line N9 was used to determine the effects of *E. africana* on inflammation *in vitro*. The cells were cultured in RPMI medium (Life Technologies) containing penicillin (100U/ml), and 10% fetal bovine serum. Cells were cultured at 37°C in a humidified incubator at an atmosphere of 5% CO_2._ N9 cells were grown in 12-well plates at a density of approximately 1 × 10^5^ cells per well. The plant compounds were dissolved in dimethylsulfoxide (DMSO) and filtered through 0.45 micrometer cellulose membranes.

### MTT assay for measuring cell proliferation

The cytotoxic effect of Ea5 was evaluated by a MTT assay. 3-(4, 5- dimethylthiazol-2-yl) -2, 5- diphenyltetrazolium bromide (MTT) is a pale yellow substrate that is reduced by living cells to yield a dark blue formazan product. This process requires active mitochondria, and only dead cells do not reduce significant amounts of MTT. Cells were seeded in 12-well plates (1 × 10^5^ cells/well) and incubated for 24 h. After this incubation period, cells were treated with various concentrations of *E. africana* (0.01, 0.1, 1, 10, and 100 μg/ml), Baicalin (5 μg/ml) and LPS (1 μg/ml) at 37°C in 5% CO_2_ for 24 h. After treatment, 100 μL of MTT (5 mg/ml) dissolved in RPMI was added to each well, followed by incubation for 3 h. The medium was aspirated, and the formazan crystals were dissolved in 500 μL of DMSO for 15 min. The optical density of each well was measured at 540 nm in a microplate reader.

### Determination of nitric oxide production

Production of NO was determined by measuring the accumulated level of nitrite, an indicator of NO in the supernatant after 24 h of LPS treatment with or without different concentrations of plant material and Baicalin. After pre-incubation of cells (1 ×10^5^ cells) for 24 h, Baicalin, or Ea5 (0.05, 0.5, 5, and 50 μg/ml) were added, together with LPS (1 μg/ml). The cells were further incubated at 37°C, 5% CO_2_ for 24 h. The quantity of nitrite in the culture medium was measured as an indicator of NO production. Amounts of nitrite, a stable metabolite of NO, were measured using Griess reagent (1% sulfanilamide and 0.1% naphthyl ethylene diamine dihydrochloride in 2.5% phosphoric acid). Briefly, 50 μl of cell culture medium was mixed with 100 μl of Griess reagent. Subsequently, the mixture was incubated at room temperature for 10 min and the absorbance at 570 nm was measured in a microplate reader. Fresh culture medium was used as a blank in every experiment. The quantity of nitrite was determined from a sodium nitrite standard curve.

### RNA Extraction and Reverse Transcription-Polymerase Chain Reaction (RT-PCR)

N9 cells were treated with Baicalin, Ea5 and LPS (1 μg/ml) for 24 h. Total RNA from LPS-treated N9 cells was prepared using the innuPREP RNA Mini kit (QIAGEN GmbH, Hilden, Germany) according to the manufacturer’s protocol. cDNA (1 μg/ml) was synthesized from 1 μg of total RNA and was used to perform RT-PCR. After initial denaturation for 2 min at 95°C, thirty amplification cycles were performed for iNOS (1 min of 95°C denaturation, 1 min of 60°C annealing, and 1.5 min 72°C extension), TNF-α (1 min of 95°C denaturation, 1 min of 55°C annealing, and 1 min 72°C extension), IL-1β (1 min of 94°C denaturation, 1 min of 60°C annealing, and 1 min 72°C extension), IL-6 (1 min of 94°C denaturation, 1 min of 60°C annealing, and 1 min 72°C extension) and β-actin (1 min of 94°C denaturation, 1 min of 60°C annealing, and 1 min 72°C extension). The primer sequences used for quantification of iNOS, TNF α, IL-1 β, IL-6, β-actin and the PCR conditions are described in the Table 1. PCR products were separated by 1.5% agarose gel electrophoresis containing 10 mg/ml ethidium bromide, photographed using UVsolo system (Whatman Biometra, Goettingen, Germany) and densitometric analysis was performed with the software BioDocAnalyze (Whatman Biometra). Results were calculated as levels of target mRNAs relative to those of β-actin.

**Table 1:** Primers used for RT-PCR analysis (F: forward, R: reverse)

**Gene**	**Primer sequences**
TNFα	F 5′-TTGACCTCAGCGCTGAGTTG-3′
	R 5′-CCTGTAGCCCACGTCGTAGC-3′
IL-1β	F5′-CAGGATGAGGACATGAGGCACC-3′
	R 5′-CTCTGCAGACTCAAACTCCAC-3′
IL6	F5′-GTACTCCAGAAGACCAGAGG-3′
	R 5′-TGCTGGTGACAACCACGGCC-3′
iNOS	F5′-CCCTTCCGAAGTTTCTGGCAGCAGC-3′
	R5′-GGCTGTCAGAGCCTCGTGGCTTTGG-3
β-actin	F5′-CCGTCTTCCCCTCCATCGT-3′
	R5′-ATCGTCCCAGTTGGTTACAATGC-3′

### p38 MAP Kinase inhibition assay

The inhibition of p38 MAP kinase was realized as described by [20]. Briefly, 96-well plates were coated with ATF-2, overnight, at 4°C. Blocking buffer was added at room temperature and the plates were incubated for 30 min before the addition of the kinase reaction mix containing the enzyme, with or without test compounds. The p38α reaction was carried out by using kinase (12 ng per well), ATP (100 μM), for 45 min at 37°C. The ATF-2 phosphorylation was detected with a specific anti-phospho ATF-2 (Thr69/71) antibody (60 min at 37°C). After each incubation time, the plate was washed three times with double distilled water. The optical density was measured after the addition of the substrate at 450 nm, by a plate reader. The p38 MAPK Kinase inhibitor SB203580 was used as a reference compound.

### Statistical analysis

All experiments were reiterated at least three times in triplicate. The results of multiple observations are expressed as the mean ± SD. Statistical significance was determined by one-way analysis of variance (ANOVA) using Graph Pad Prism 5.0 for windows. For all statistical analyses, significance levels were set at *P* < 0.05.

## Results

### Effect of *Entada africana* on the viability of N9 cells

**Figure 1:**
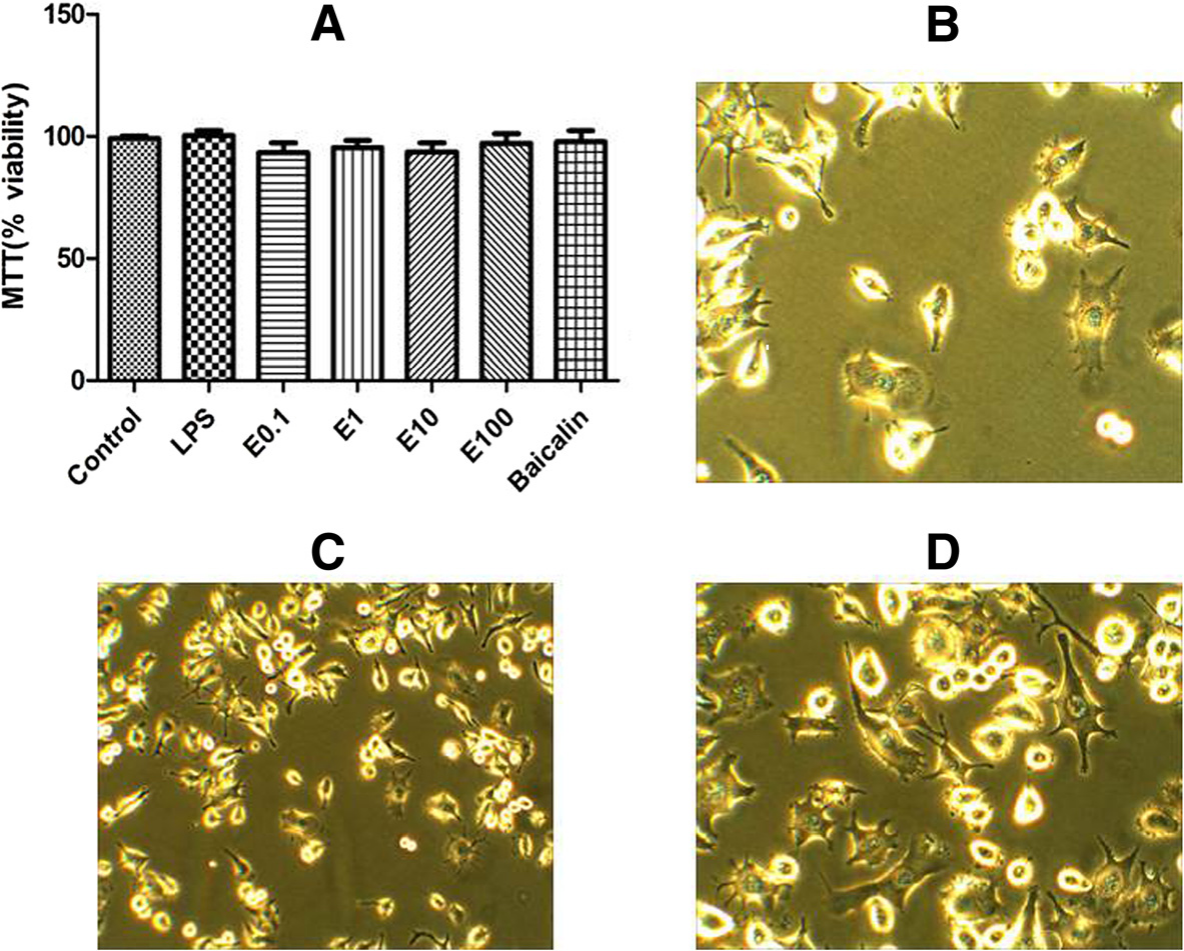
**Effect of *E. africana *and Baicalin on the viability of LPS-activated N9 cells. (A)** N9 cells 1x10^5^cells/ml were incubated with the indicated concentrations of *E. africana* and Baicalin (5 μg/ml) in the presence of LPS 1 μg/ml for 24 h. The cell viability was then determined by MTT assay as described in the Methods. **(B)**: N9 cells incubated with DMSO for 24 h **(C)**: N9 cells incubated with LPS (1 μg/ml) and *E. africana* (50 μg/ml) for 24 h. **(D)**: N9 cells incubated with LPS (1 μg/ml) and Baicalin (5 μg/ml) for 24 h.

Examination of the cytotoxicity of Ea5 on N9 cells by MTT assay indicated that these compounds, even at 100 μg/ml, did not affect the viability of N9 cells (Figure 1).

### Ea5 Inhibition of LPS-induced NO production in N9 microglia

**Figure 2:**
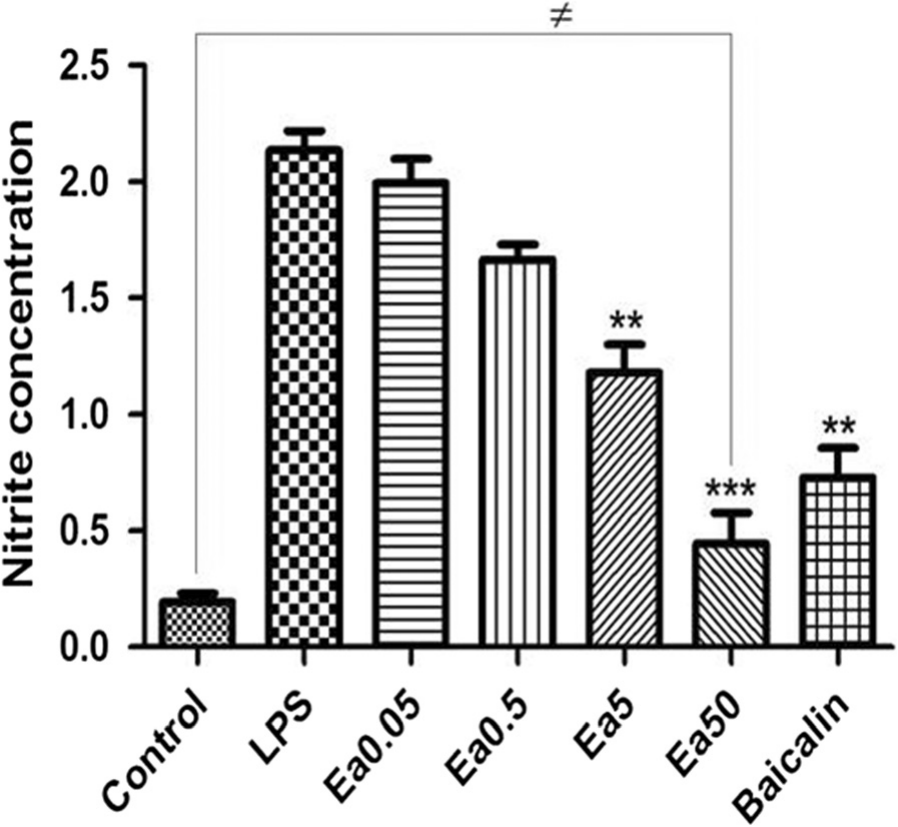
**Inhibition of LPS-induced production of Nitric Oxide by *E. africana*.** The extract was prepared according to the protocol and added to cultured N9 cells together with LPS at 1 μg/ml. The plant extract was applied at final concentrations ranging from 0.05 to 50 μg/ml. Baicalin was used at 5 μg/ml. Following 24 h incubation, cell supernatant was collected and NO was quantified as described in Methods. * *P* < 0.05, **P < 0.01 and ***P < 0.001 compared to LPS treated group. ≠ indicate no significant difference between the control group and Ea50 treated group. Error bars indicate standard deviation.

The effects of Ea5 on LPS-induced NO production in N9 microglia were investigated by measuring the accumulated nitrite, as estimated by the Griess reaction in the culture medium. After treatment with LPS (1 μg/ml) for 24 h, nitrite concentrations in the medium increased remarkably by approximately 10 fold. When N9 microglia were treated with different concentrations of the indicated compounds together with LPS (1 μg/ml) for 24 h, a significant (P < 0.001) concentration-dependent inhibition of nitrite production was detected in the presence Ea5 (Figure 2).

### Effects of Ea5 on pro-inflammatory cytokines and iNOS mRNA expression

**Figure 3:**
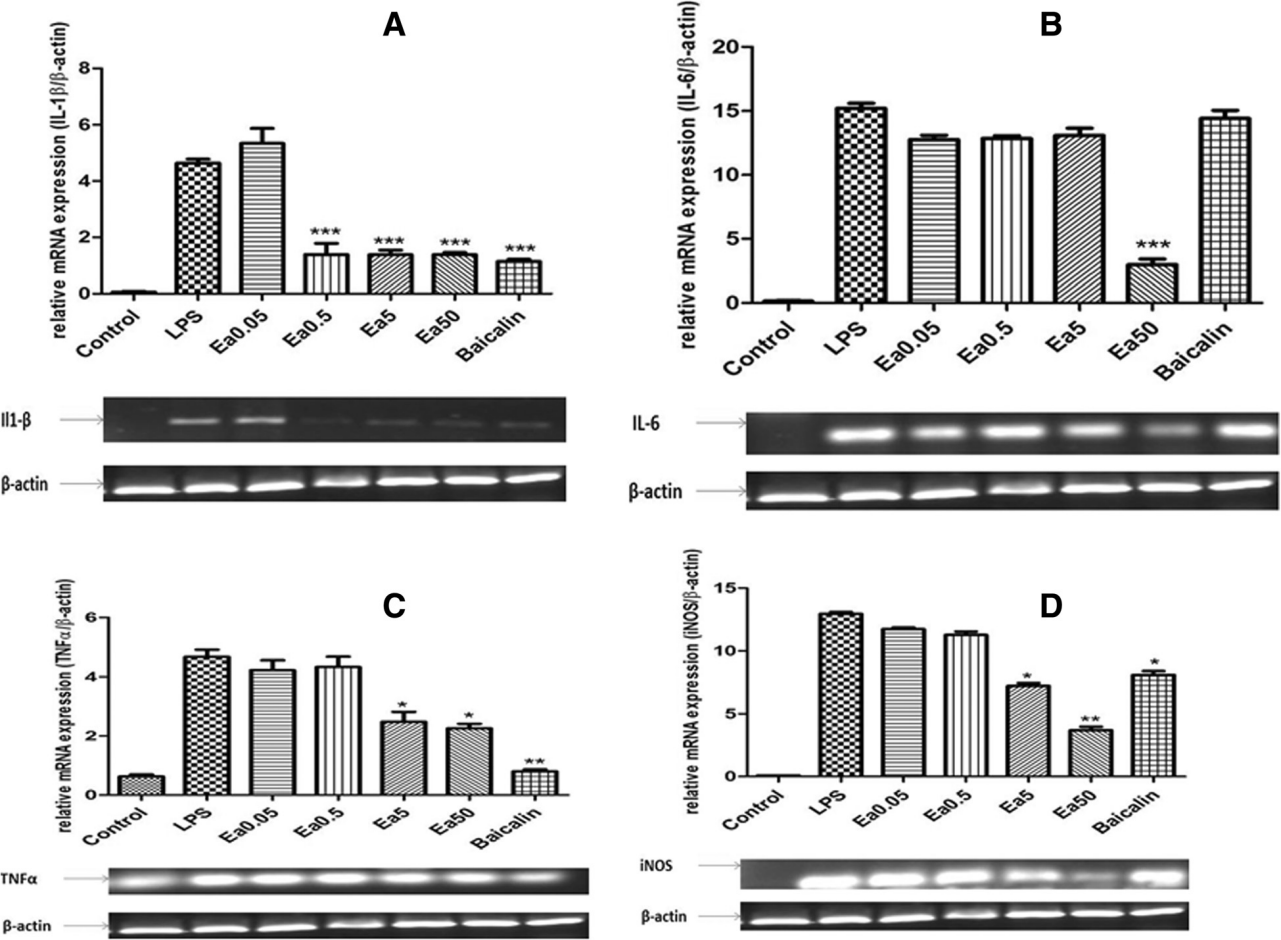
**Effect of *E. africana *fraction CH**_**2**_**Cl**_**2**_**/MeOH 5% on LPS-induced expression of proinflammatory cytokines and iNOS in microglia.** N9 cells were pre-treated with different concentrations of Ea5 (0.05, 0.5, 5 and 50 μg/ml) and or Baicalin (5 μg/ml), with or without LPS for a stimulation period of 24 h. Total RNAs were isolated, and mRNA levels of IL1β, IL6 and TNFα were measured by RT-PCR. β-actin expression was used as an internal control. **(A)**. IL1β, **(B)**. IL6 and **(C)**. TNFα.* *P* < 0.05, **P < 0.01 and ***P < 0.001 compared to LPS treated group. **(D)**: *E. africana* fraction CH_2_Cl_2_/MeOH 5% and Baicalin reduced LPS-induced iNOS gene expression in macrophage cell line. N9 cells were incubated without or with LPS 1 μg/ml together with the indicated amounts of the extract or Baicalin. After 24 hours iNOS mRNA was quantified by qRT-PCR.* *P* < 0.05, **P < 0.01 and ***P < 0.001 compared to LPS treated group.

We next investigated whether Ea5 suppresses the production of pro-inflammatory cytokines, such as IL-6, IL-1β and TNFα in LPS-stimulated microglia cells. For this study, N9 microglias were incubated with Ea5 and Baicalin in the presence or absence of LPS for 24 h, and the cytokines mRNA expressions were evaluated. As shown in Figure 3A, B and C, 1 μg/ml LPS significantly increased IL1-β, IL-6 and TNFα mRNA levels in cells of the microglia cell line N9. *E. africana* significantly inhibited IL-6 mRNA expression at 50 μg/ml (P < 0.001). A significant inhibition of IL-1β was obtained with Ea5 at 0.5, 5 and 50 μg/ml (P < 0.001). The same effect was observed with Baicalin. A minor but significant inhibition of the mRNA expression of TNFα was obtained at 5 and 50 μg/ml (P < 0.05), whereas a significant inhibition was obtained with Baicalin at the tested concentration (P < 0.01).

We carried out RT-PCR to investigate the question whether inhibition of NO production was associated with decreased levels of iNOS expression. As shown in Figure 3D, 1 μg/ml LPS significantly increased iNOS mRNA levels in these cells after 24 h treatment. Ea5 significantly reduced the mRNA expression of iNOS at 50 μg/ml (P < 0.01). An inhibition of iNOS was also observed at 5 μg/ml and with Baicalin (P < 0.05).

### Effect of Ea5 on p38 MAPK Kinase inhibition

**Figure 4:**
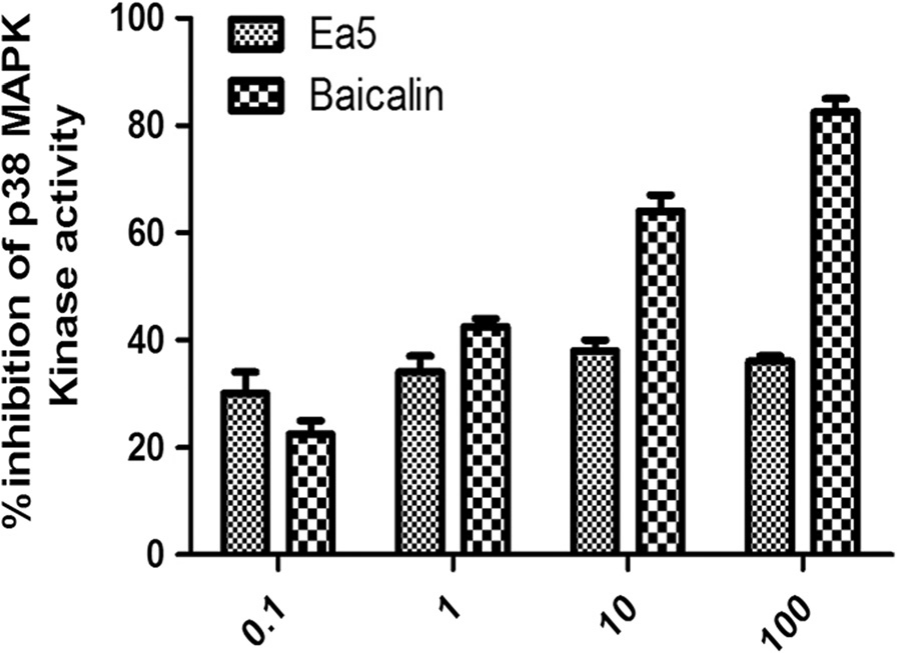
**Effect of *E. africana *fraction CH**_**2**_**Cl**_**2**_**/MeOH 5% and Baicalin on the inhibition of p38 MAPK Kinase activity.** The inhibitory potency of the fraction and Baicalin was evaluated by using p38α ELISA assay containing BSA (0.01%) in the kinase buffer. SB203580 was used as reference compound.

MAPKs constitute a family of specific serine/threonine kinases that phosphorylate target substrates, thereby control important cellular functions such as gene expression [21]. Ea5 and Baicalin were tested for their ability to inhibit p38MAPK Kinase. This was evaluated by using a p38α ELISA assay containing BSA (0.01%) in the kinase buffer. In our experiment, the p38 MAPK inhibitor SB203580 (IC50 = 0.048+/- 0.01 μM) was used as a reference compound for the p38α kinase assay. Ea5 showed a moderate inhibitory effect on the enzyme over the concentration range tested (0.1 to 100 μg/ml). Baicalin inhibited the activity of p38 MAPK Kinase up to 80% (Figure 4).

## Discussion

Inflammation plays an important role in the pathology of neurodegenerative disorders in the brain [22]. In particular, neuroinflammation with prolonged activation of microglia cells leads to an increased activation of these cells and to increased production of proinflammatory mediators and cytokines [23], contributing to neuronal dysfunction and neuronal cell death. Therefore, inhibitors of these inflammatory molecules have been considered as candidate anti-inflammatory drugs for alleviating the progression of neurodegenerative diseases caused by activation of microglia. The expression of iNOS and the overproduction of NO are considered to play a significant role in the pathogenesis of various neurodegenerative diseases. For instance, overproduction of NO by microglia contributes to the complication of AD [24] and Parkinson’s disease [25].

In this study, we explored for the first time the effect of *E. africana* fraction CH_2_Cl_2_/MeOH 5% on NO production as well as on the expression of pro-inflammatory cytokines and iNOS mRNA in microglia cells *in vitro*.

In comparison to our recent study on the effect of Ea5 on Raw 264.7 cells, the inhibition of NO production was 89.06% with Raw 264.7 cells (and 87.07% for N9 cells). Baicalin presented an inhibition of NO production of 63.34% and 70.85% respectively for Raw cells and N9 cells. After treatment with LPS (1 μg/ml) for 24 h, nitrite concentration in the medium was not significantly different in Raw 264.7 cells (2.011 ± 0.027 μM) and in N9 cells (2.2 ± 0.079 μM). Concerning the inhibition of proinflammatory cytokines, Ea5 significantly reduced the mRNA expression of IL-6, TNFα and IL-1β respectively in Raw 264.7 cells and in N9 cells respectively.

Concerning the inhibition of NO, Ea5 presented the highest inhibition (87.07%) in comparison with Baicalin (70.85%). An inhibition of the mRNA expression of IL1-β and IL-6 was obtained with Ea5 and Baicalin respectively. Concerning the inhibition of iNOS mRNA expression, a significant inhibition was obtained with Ea5 (*P* < 0.01) and with Baicalin (*P* < 0.05). These results suggest that Ea5 may have at least the same anti-inflammatory potency as Baicalin.

*E. africana* has been reported to be a potent antioxidant with scavenging activities and to induce hemeoxygenase-1 expression, similar to most antioxidants [16]. The data presented here suggest that *E. africana* can strongly protect host immune cells from inflammation-mediated cytotoxic conditions induced by cytokine production and NO generation.

Cytokine produced by microglia have been shown to be intimately associated with amyloid deposits and have also been implicated as scavengers responsible for clearing Aβ deposits [26]. Although the molecular mechanism by which *E. africana* inhibits the production of NO and pro-inflammatory cytokines mRNA expression has not been elucidated, *E. africana* may modulate a common pathway of microglia response. To date, several important common pathways have been identified. One of these pathways is known to be the nuclear factor NF-kappaB, as it controls the expression of various protein markers such as cytokines and surface glycoproteins. Anti-inflammatory effects of many plant derived compounds have also been reported to occur via inhibition of the MAPKs, PI3K/Akt, and Jak/STAT pathways [27].

Due to the antioxidant properties of *E. africana*[16], we presumed that the suppressive effect of this plant extract on NO production may be due to its scavenging properties and the suppression of iNOS at the transcriptional level.

*E. africana* main constituents are polyphenols [16]. The phenolic compounds present in this plant extract may be responsible for the observed effects, as many polyphenols have in many reports been reported as good anti-inflammatory and immunomodulatory compounds [28,29]. The results obtained in this study indicate that, *E. africana* may also be a good candidate for the treatment of neuropathologies and may act by inhibiting the over-activation of microglias besides its antioxidant activity.

However, there are some limitations in the present study. The study does not evaluate the effect of the extract on the expression of cytokines, iNOS and NFkB proteins by western blot. Moreover, many other inhibitors such as COX-2 inhibitors should be used as positive controls to evaluate the effect of the extract on prostaglandins production and COX-2 expression. These limitations will therefore be investigated in our future studies.

## Conclusion

The present study has revealed that *E. africana* treatment of mouse microglia cells inhibited LPS-induced NO production by suppressing iNOS mRNA expression. *E. africana* also inhibited the production of pro-inflammatory cytokines, such as TNFα, IL-6 and IL1-β, by suppressing their transcriptional activity, the same as did Baicalin, the reference compound used in this study. This suggests that *E. africana* may have substantial therapeutic potential for treatment of neurodegenerative diseases that are accompanied by microglia activation. Ongoing studies concentrate on the isolation and determination of the pure active compounds present in Ea5 and explore the signaling pathways responsible for the observed effects.

## Abbreviations

Aβ: Amyloid beta; Ea5: *Entada africana* fraction CH_2_Cl_2_/MEOH 5%; LPS: Lipopolysaccharide; NFkB: Nuclear factor kappaB.

## Competing interests

The authors declare that they have no competing interests.

## Authors’ contributions

VBOA carried out the study design, cell culture, the experiments, literature search and manuscript preparation. HS and SL carried out the cell culture and biochemical experiments. NFN and Paul FM collected the plants and contributed in drafting the manuscript. All authors read and approved the final manuscript.

## Pre-publication history

The pre-publication history for this paper can be accessed here:

http://www.biomedcentral.com/1472-6882/13/254/prepub
